# A high mortality rate associated with multidrug-resistant *Acinetobacter baumannii* ST79 and ST25 carrying OXA-23 in a Brazilian intensive care unit

**DOI:** 10.1371/journal.pone.0209367

**Published:** 2018-12-28

**Authors:** Kesia Esther da Silva, Wirlaine Glauce Maciel, Julio Croda, Rodrigo Cayô, Ana Carolina Ramos, Romário Oliveira de Sales, Mariana Neri Lucas Kurihara, Nathalie Gaebler Vasconcelos, Ana Cristina Gales, Simone Simionatto

**Affiliations:** 1 Laboratório de Pesquisa em Ciências da Saúde, Universidade Federal da Grande Dourados—UFGD, Dourados—Mato Grosso do Sul, Brazil; 2 Fundação Osvaldo Cruz—FIOCRUZ, Campo Grande—Mato Grosso do Sul, Brazil; 3 Faculdade de Medicina, Universidade Federal da Grande Dourados—UFGD, Dourados—Mato Grosso do Sul, Brazil; 4 Universidade Federal de São Paulo—UNIFESP, Laboratório Alerta, Disciplina de Infectologia, Departamento de Medicina, Escola Paulista de Medicina—EPM, São Paulo—SP, Brazil; University of Georgia, UNITED STATES

## Abstract

The global spread of carbapenem-resistant *Acinetobacter baumannii* (*A*. *baumannii)* strains has restricted the therapeutic options available to treat infections due to this pathogen. Understanding the prevalence of such infections and the underlying genetic mechanisms of resistance may help in the implementation of adequate measures to control and prevent acquisition of nosocomial infections, especially in an intensive care unit setting. This study describes the molecular characteristics and risk factors associated with OXA-23-producing *A*. *baumannii* infections. A case-control study was undertaken from September/2013 to April/2015. Acquisition of OXA-23-producing *A*. *baumannii* was found to be associated with the use of nasogastric tubes, haemodialysis, and the use of cephalosporins. These isolates were only susceptible to amikacin, gentamicin, tigecycline, and colistin, and contained the IS*Aba1* insertion sequence upstream of*bla*_OXA-23_ and *bla*_OXA-51_ genes. Twenty-six OXA-23-producing *A*. *baumannii* strains belonged to the ST79 (CC79) clonal group,and patients infected or colonised by these isolates had a higher mortality rate (34.6%). In conclusion, this study showed a dissemination of OXA-23-producing *A*. *baumannii* strains that was associated with several healthcare-related risk factors and high mortality rates among intensive care unit patients.

## Introduction

The spread of carbapenem-resistant *Acinetobacter baumannii* strains has been reported worldwide over recent decades, and has become a matter of great concern[1].This pathogen can cause a variety of diseases associated with high morbidity and mortality rates and can be difficult to treat due to its multidrug resistance (MDR) phenotype. In addition, this pathogen has the potential to spread and maintain itself within healthcare facilities [2].

Carbapenem resistance rates among *A*. *baumannii* strains have increased considerably in Latin American countries. The main mechanism responsible for this phenotype is the acquisition of genes encoding class D carbapenemases or oxacillinases. Such isolates have become a significant threat to the control and treatment management of nosocomial infections, and have been commonly associated with hospital outbreaks[3].The carbapenemases belonging to the groups OXA-23, OXA-24/40, OXA-58, and OXA-143are frequently observed among *A*. *baumannii* clinical isolates[4]. The genes encoding for such enzymes are generally associated with mobile genetic elements, such as insertion sequences (IS) that lead to their mobilization for other *A*. non-*baumannii* species[5–7].

The presence of IS, predominantly IS*Aba1*, upstream of oxacillinase-encoding genes is associated with an increased gene expression leading to a resistance to carbapenems[8]. Several outbreaks due to OXA-23-producing *A*. *baumannii* clones have previously been described[1,9,10],and these strains are an international public health concern[3]. Here, we performed a case-control study in an adult intensive care unit (ICU) in a tertiary teaching hospital in Brazil to identify the risk factors associated with OXA-23-producing *A*. *baumannii* isolates.

## Materials and methods

### A case-control study

To identify risk factors associated with the acquisition of OXA-23-producing *A*. *baumannii* infections, a case-control study was conducted in two adult ICUs in a tertiary teaching hospital located in Dourados city, Mato Grosso do Sul (a central-western Brazilian state). We included patients hospitalised from September/2013 to April/2015 in this study. A case was defined as an adult patient who presented with OXA-23-producing *A*. *baumannii* strains isolated from clinical specimens from any source during the study period. Controls were patients from whom no *A*. *baumannii* was isolated within the first 48 hours after admission[9]. For each case, a respective control was selected from adult inpatients admitted within the study period matched for age, clinical manifestation, and hospital ward. All medical, nursing, and microbiological records of hospitalised patients were reviewed. We reviewed inpatient clinical records, and the following data were recorded: demographics, medical histories and co-morbid conditions, previous hospitalisation, ward of admission, hospital course (length of stay, and hospital ward), invasive procedures (devices used, and surgery), mechanical ventilation, treatment with immunosuppressive drugs, antibiotic exposure history, source of infection (blood, urinary tract, wound, respiratory source, or other), and clinical outcome (recovery or death).

All comorbidities were evaluated including diabetes mellitus, cardiovascular disease, renal failure, respiratory failure, chronic obstructive pulmonary disease, alcoholism, smoking history, neoplasia, neurological disease, sepsis, use of illicit drugs, HIV infection, decubitus ulcers, cancer, and hypertension. All antibiotics administered for ≥24 hours during the current hospitalisation period were recorded. The information collected included the drug name, start date, dose, route of administration, dosing frequency, and total duration of use. Both the individual and cumulative antibiotic exposures were also evaluated.

### Bacterial strains

The OXA-23-producing *A*. *baumannii* strains were obtained from 41 different adult patients, and isolates were collected on different days and from different body sites. Colonisers were defined as bacteria that were either permanently or temporarily present in the skin or mucous membranes of the host, and which were not associated with the symptoms and/or presence of signs of clinical infection. Clinical infection was defined as isolation of OXA-23-producing *A*. *baumannii* isolates in addition to a medical diagnosis, according to the clinical criteria (sepsis, fever, changes in frequency or colour of secretions, or new radiological findings), associated with the decision to initiate antibiotic therapy[11]. The study was conducted with the approval of the Research Ethics Committee from the Universidade Federal da Grande Dourados- UFGD (number 877.292/2014) who determined that a waiver of informed consent was appropriate.

### Bacterial identification, susceptibility testing, and phenotypic assays

Bacterial species identification was performed using the automated system Vitek2 (bioMérieux, Hazelwood, MO) and confirmed with matrix-assisted laser desorption ionization-time of flight mass spectrometry (MALDI-TOF MS), using a Microflex LT spectrometer (BrukerDaltonics, MA, USA), as previously described[12]. The minimal inhibitory concentrations (MICs) of antimicrobials were determined using Vitek2 for the following drugs:ampicillin/sulbactam,piperacillin/tazobactam,ceftazidime, ceftriaxone, cefepime, imipenem, meropenem, gentamicin, ciprofloxacin, colistin, and tigecycline. The tigecycline MICs were confirmed using Etest strips (bioMerieux Marcy l’Étoile, France) according to the manufacturer’s recommendations. Susceptibility results were interpreted according to the breakpoints recommended by the Clinical and Laboratory Standards Institute (CLSI)[13], except for tigecycline for which there is no breakpoint available for *A*. *baumannii*[14]. Preliminary screening for the production of carbapenemases was performed usingan ertapenem hydrolysis assay (2 and 4 hours) using MALDI-TOF MS, as previously described[15]. Carbapenem hydrolysis was considered positive if the ertapenem intact-molecule mass peak (475 m/z), and that of its monosodium salt (497 m/z), disappeared completely[15,16].

### Polymerase chain reaction (PCR) amplification

The presence of genes encoding beta-lactamases (*bla*_CTX-_like, *bla*_GES-_like, *bla*_SHV-_like,*bla*_IMP-_like, *bla*_NDM-_like, *bla*_VIM-_like, *bla*_KPC-_like, *bla*_OXA-23-_like, *bla*_OXA-24/40-_like, *bla*_OXA-48-_like, *bla*_OXA-51-_like, *bla*_OXA-58-_like, and *bla*_OXA-143-_like)[17,18], as well as the presence of IS*Aba1* upstream of oxacillinase encoding genes, was evaluated using PCR followed by sequencing using specific primers, as previously described[8,19]. The DNA sequences and their derived protein sequences were analysed using the Lasergene Software Package (DNASTAR, Madison, USA), and compared with sequences deposited in GenBank.

### Molecular typing using pulsed-field gel electrophoresis (PFGE)

The genetic relationship among the OXA-23-producing *A*. *baumannii* strains was determined with PFGE using the restriction enzyme *Apa*I (New England BioLabs, Ipswich, MA, USA). The restriction fragments were separated on a 1% (w/v) agarose gel in a 0.5% tris-borate-EDTA buffer in a CHEF-DR II electrophoresis system (Bio-Rad Laboratories, Richmond, CA, USA) for 19 h at 14°C, using a pulse ramp rate changing from 5s to 60s, at 6 V/cm.The restriction patterns were analysed using BioNumerics software v. 6.0 (Applied Maths, Sint-Martens-Latem, Belgium). Percentage similarity between fingerprints was scored using the Dice coefficient[20]. Theunweighted pair group method with arithmetic mean method was used to construct the dendrogramwith a 1.5% tolerance limit.

### Multilocus Sequence Typing (MLST)

MLST analysis was performed according to the Institute Pasteur scheme through double-stranded DNA sequencing of the internal regions of seven housekeeping genes (*cpn60*, *fusA*, *gltA*, *pyrG*, *recA*, *rplB*, and *rpoB*). Determination of the sequence type (ST) was performed through the *A*. *baumannii* MLST (Pasteur) database (http://pubmlst.org/abaumannii/bigsdb?db=pubmlst_abaumannii_pasteur_seqdef). The relationship between new and existing STs was assessed using the eBURST program(http://eburst.mlst.net/)[21,22].

### Statistical analysis

All clinical data were deposited in the Research Electronic Data Capture (Redcap) database and statistical analysis was performed with SAS v. 9.2 (SAS Institute, Cary, NC, USA), using univariate and multivariate models. Dichotomised and categorical data were analysed using chi-square or Fisher’s exact tests. For continuous variables, a *t*-test or ANOVA was used. Bivariate analyses were performed to verify the associations between dependent and independent variables, and those achieving a pre-specified level of significance (*P*<0.05) were included in the multivariable analysis. Logistic regression analysis was used to estimate the crude and adjusted odds ratios (OR). The calculation of the endemic level of colonisation or infection per 1,000 patient-days was calculated according to the method described previously[23].

## Results

During the study period a total of 275 episodes of *A*. *baumannii* infection/colonisation were observed. Of these, 41 (15%) OXA-23-producing strains were recovered from patients hospitalised in adult ICUs. The ages of the 41 patients colonised or infected due to OXA-23-producing *A*. *baumannii* isolates ranged from 24 to 87 years, with 46.3% of the patients aged >60 years old. Most patients were male (n = 24 of41, 58.5%). The patients presented with multiple comorbidities such as diabetes mellitus, hypertension, chronic diseases, and sepsis. Additionally, they had undergone many invasive procedures such as mechanical ventilation, prior surgery, the use of a nasogastric tube, and central venous and urinary catheters. The length of hospitalisation was >15 days for most patients (n = 27 of 41,65.8%) with a past history of hospitalisation (92.6%). Prior to isolation of OXA-23-producing *A*. *baumannii*, all patients received antimicrobial therapy over a period of up to 30 days that included penicillin, third-or fourth-generation cephalosporins, aminoglycosides, fluoroquinolones, amikacin, tigecycline, piperacillin/tazobactam, trimethoprim, carbapenems, and polymyxins.

**Fig 1:**
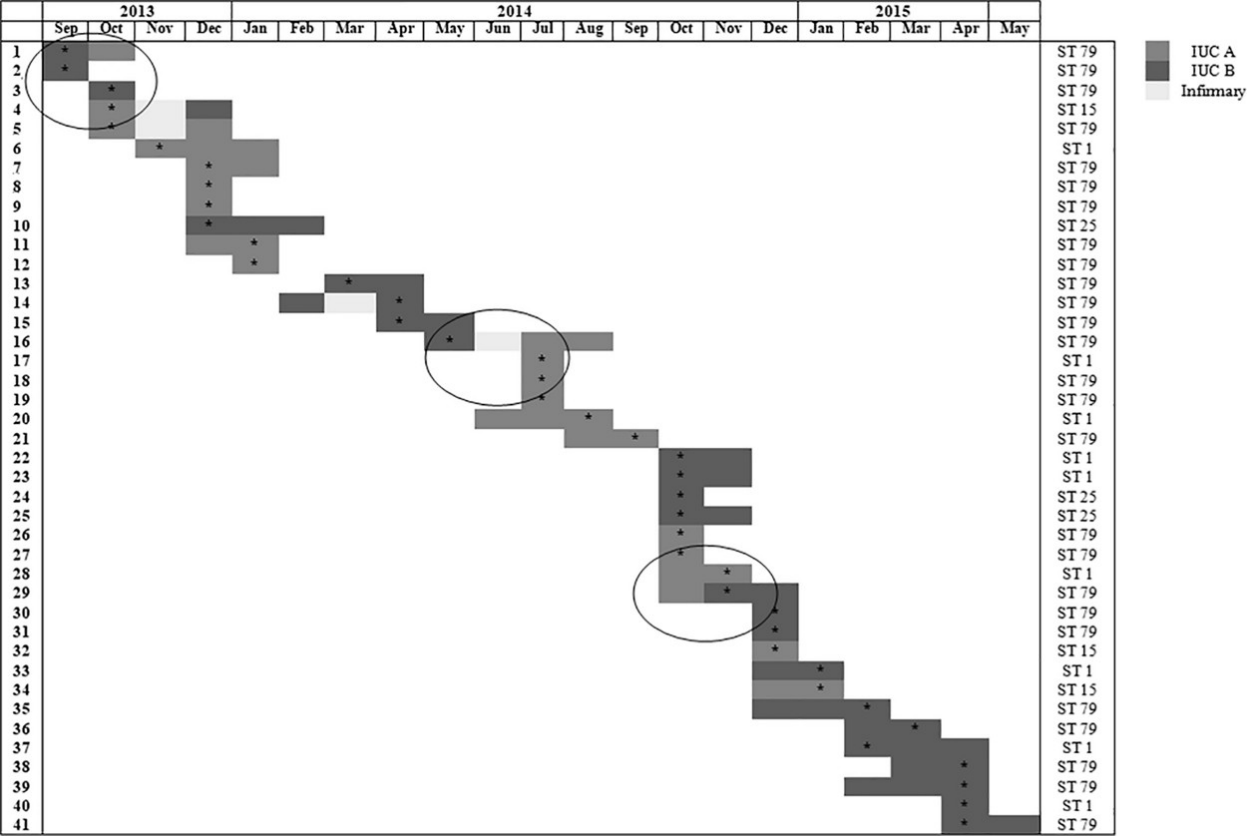
Time-line of events during the patient admission period. The isolation of OXA-23-producing *A*. *baumannii* strains are marked with an asterisk. The arches highlight patients who moved to a different ICU during hospitalisation.

**Fig 2:**
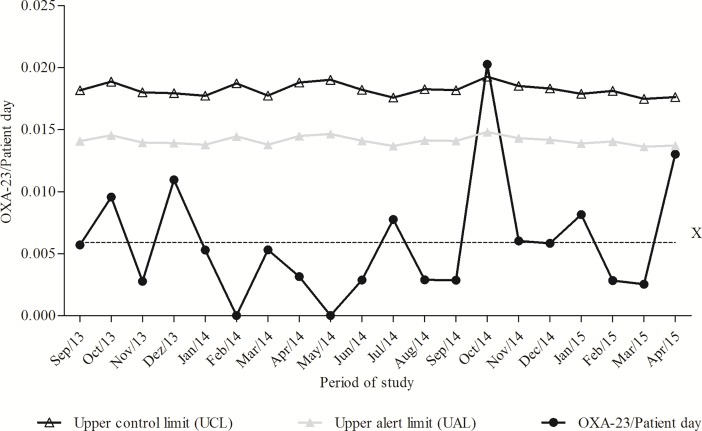
Temporal distribution of OXA-23-producing *A*. *baumannii* strains in the ICU per patient-days from September 2013 to April 2015. **X** centre line (average rate of OXA-23 infections per patient-days = 0.0058).

The 41 OXA-23-producing *A*. *baumannii* strains were identified in the patients between 1 and 95 days after admission. The time-line of events during the inpatient admission period, including the stay on the wards, and OXA-23-producing *A*. *baumannii* strains isolation, is illustrated in Fig 1. Four patients moved from different ICUs during hospitalisation. Most OXA-23-producing *A*. *baumannii* (n = 30, 73.2%) were considered true pathogens, and were mainly recovered fromtracheal aspirate cultures (n = 23, 56.1%), followed by nasal and rectal swabs (n = 11, 26.8%), wounds (n = 3, 7.3%), blood (n = 2, 4.9%), and urine (n = 2, 4.9%). From September2013 to April2015, the mean rate of OXA-23-producing *A*. *baumannii* strains was 5.8 isolates per 1,000 patient-days. However,this rate exceeded the control limit established, and led to a mean incidence of 20 isolates per 1,000 patient-days in October2014, falling to 6 isolates per 1,000 patient-days in November 2014 (Fig 2).

The case-control study comprised 82 patients (41 cases, 41 controls). No significant differences (*P* > 0.05) were observed between both groups regarding baseline demographic data. In a multivariate analysis, OXA-23-producing *A*. *baumannii* strains were associated with the use of nasogastric tubes, haemodialysis, and therapy with cephalosporins (Table 1). The analysis of patient outcomes revealed that patients with OXA-23-producing *A*. *baumannii* isolates had a high mortality rate (n = 14, 34.1%).

**Table 1:** Summary of risk factors associated with OXA-23-producing *A*. *baumannii* strains.

Variables	Case patients (n = 41)	Control patients (n = 41)	Univariable analysis		Multivariable analysis	
			OR (95% CI)	P-value	OR (95% CI)	P-value
Age (years)	63. 84±15.42	61.74±15.14				
**Comorbidities**						
Diabetes mellitus	16 (39.02)	8 (19.51)	2.64 (0.97–7.14)	0.05		
Alcoholism	8 (19.51)	6 (14.63)	1.41 (0.44–4.51)	0.55		
Hypertension	24 (58.53)	21 (51.22)	1.3 (0.56–3.21)	0.50		
Decubitus ulcers	6 (14.63)	3 (7.32)	2.17 (0.50–9.35)	0.28		
Pulmonary disease	2 (4.88)	8 (19.51)	0.21 (0.04–1.06)	0.04		
Chronic heart failure	6 (14.63)	8 (19.51)	0.70 (0.22–2.25)	0.55		
Chronic renal failure	14 (34.15)	8 (19.51)	2.13 (0.78–5.85)	0.13		
Chronic respiratory failure	12 (29.27)	16 (39.02)	0.64 (0.25–1.62)	0.35		
Cancer	5 (12.20)	3 (7.32)	1.75 (0.39–7.90)	0.45		
Substanceabuse	10 (24.39)	10 (24.39)	1 (0.36–2.74)	1.00		
Neurological disease	12 (29.27)	5 (12.20)	2.97 (0.94–9.43)	0.05		
Neoplasia	8 (19.51)	3 (7.32)	3.07 (0.75–12.53)	0.10		
HIV infection	2 (4.88)	1 (2.43)	2.05 (0.17–23.54)	0.55		
Sepsis	23 (56.10)	12 (29.27)	3.08 (1.23–7.69)	0.01		
**Riskfactors**						
Mechanicalventilation	33 (80.49)	32 (78.05)	1.16 (0.39–3.38)	0.78		
Previous surgery	28 (68.29)	19 (46.34)	2.49 (1.01–6.13)	0.04		
Central venous catheter	29 (70.73)	27 (65.85)	1.25 (0.49–3.18)	0.63		
Urinary catheter	28 (68.29)	29 (70.73)	0.89 (0.34–2.28)	0.81		
Use of immunosuppressive agents	5 (12.19)	1 (2.43)	5.55 (0.61–49.82)	0.08		
Hemodialysis	**10 (24.39)**	**3 (7.32)**	**4.08 (1.03–16.15)**	**0.03**	**4.87 (1.07–22.05)**	**0.03**
Nasogastric tube	**23 (56.10)**	**10 (24.39)**	**3.96 (1.54–10.16)**	**0.00**	**4.66 (1.59–13.66)**	**<0.01**
Chest drainage	7 (17.07)	5 (12.20)	1.48 (0.42–5.12)	0.53		
Previous hospital admission	38 (92.68)	34 (82.93)	2.60 (0.62–10.89)	0.17		
**Use ofantimicrobials**						
Previous exposure	41 (100)	39 (95.12)	5.25 (0.24–112.87)	0.15		
Aminoglycosides	27 (65.85)	20 (48.78)	2.02 (0.83–4.92)	0.11		
β-lactam	20 (48.78)	12 (29.27)	2.30 (0.92–5.71)	0.07		
Carbapenems	34 (82.93)	31 (75.61)	1.56 (0.53–4.62)	0.41		
Cephalosporins	**10 (24.39)**	**26 (63.41)**	**0.18 (0.07–0.48)**	**0.00**	**6.01 (2.04–17.69)**	**<0.01**
Polymyxins	18 (43.90)	11 (26.83)	2.13 (0.84–5.38)	0.10		
Fluoroquinolones	5 (12.20)	9 (21.95)	0.49 (0.14–1.62)	0.24		

**Abbreviations:**CI, Confidence interval; HIV, Human immunodeficiency virus; OR, Odds ratio.

All OXA-23-producing *A*. *baumannii* strains were resistant to ampicillin/sulbactam (MIC_50_, ≥ 16 mg/L), piperacillin/tazobactam (MIC_50_, ≥ 128 mg/L), ceftazidime (MIC_50_, ≥ 32 mg/L), ceftriaxone (MIC_50_, ≥ 32 mg/L), cefepime (MIC_50_, ≥ 16 mg/L),imipenem (MIC_50_, ≥ 8 mg/L), meropenem (MIC_50_, ≥ 8 mg/L), gentamicin (MIC_50_, ≥ 16 mg/L), and ciprofloxacin (MIC_50_, ≥ 4 mg/L). Of 41strains,90.2%,43.9%, and 34.1% were resistant to gentamicin (MIC_50_ ≥ 8 mg/L^-1^), tigecycline (MIC_50_≥ 4 mg/L^-1^), and amikacin (MIC_50_≥ 32 mg/L^-1^), respectively. In contrast, all *A*. *baumannii* strains were susceptible to colistin (MIC_50_, ≤ 2 mg/L).The 41 strains were classified as carbapenemase producers using MALDI-TOF MS through confirmation of hydrolysis after 4 hours of incubation.

**Fig 3:**
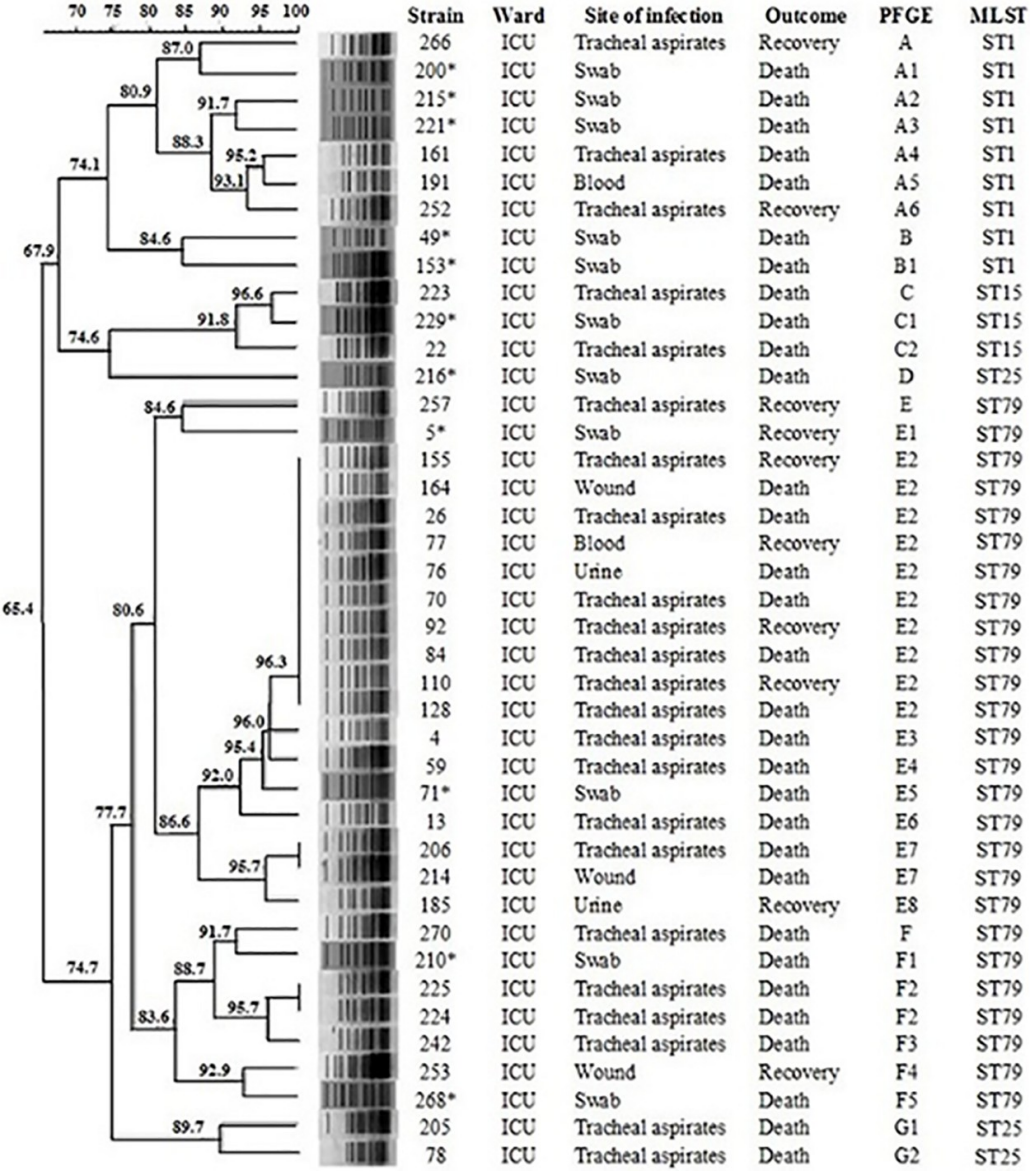
Dendrogram displaying the genetic relatedness of 41 OXA-23-producing *A*. *baumannii* strains recovered in a Brazilian ICU, based on PFGE data and MLST content. Asterisks indicate the colonizing strains.

PCR amplification and sequencing showed that the IS*Aba1* was found upstream both *bla*_OXA-23_ and *bla*_OXA-51_ genes in all carbapenem-resistant strains. None of the other beta-lactamases-encoding genes were detected. PFGE analysis of 41 OXA-23-producing *A*. *baumannii* strains identified seven clusters (Fig 3; A to G). However, 46.3% (n = 19) of isolates belonged to cluster E that, together with cluster F (n = 7),were identified as ST79 lineage (CC79; n = 26,63.4%). Most ST79 isolates (n = 22 of 26, 84.6%) caused lower respiratory tract infections diagnosed throughout the study period, demonstrating the long-term persistence of this endemic clone in the ICU. Data analysis also revealed that patients infected with this predominant clonal group showed a higher mortality rate (n = 18, 69.2%;*P*≤ 0.01) compared to the other strains. Nine isolates were identified as ST1 (CC1) (clusters A and B, 21.9%), three as ST25 (CC25) (clusters D and G, 7.3%) and three as ST15 (CC15) (cluster C, 7.3%).

After notification of the increase in the number of carbapenem-resistant *A*. *baumannii* strains in October2014, measures were taken to contain the outbreak. Infection control measures were implemented, and surveillance cultures were collected from all patients who had been hospitalised for >48 hours in key wards, particularly for patients who had been previously hospitalised. The infection control measures included individual use of medical equipment, hand hygiene promotion in the ICU, isolation of patients colonised or infected with carbapenem-resistant strains, and environmental cleaning and disinfection of reusable medical equipment. Furthermore, cleaning of all surfaces, including walls, floors, ceilings, windows, furniture, and medical equipment was intensified. Additionally, exchange of antiseptic solutions and daily summaries of all relevant microorganisms on the ICUwere performed, and communication among the medical microbiology laboratory staff, infection prevention and healthcare workers was intensified through regular educational meetings. There were no interventions on antibiotic administration, and patients with infections were treated according to the standard antimicrobial treatment policy (combination therapy with amikacin and polymyxin B).

## Discussion

OXA-23-producing *A*. *baumannii* has been increasingly reported worldwide[24,25], accounting for between 42%–100% of nosocomial carbapenem-resistant *A*. *baumannii* in Brazil and affecting severely ill patients with high morbidity and mortality rates[24,26]. Infected or colonised patients represent reservoirs for horizontal transmission and spread of multidrug-resistant *A*. *baumannii*, especially in ICUs [27,28]. Assessment of factors that predict carbapenem resistance using multivariable analysis demonstrated that nasogastric tubes, hemodialysis, and cephalosporin users were associated with OXA-23-producing *A*. *baumannii*. Haemodialysis and the use of nasogastric tubes have previously been described as risk factors for acquiring carbapenem-resistant *A*. *baumannii* in hospitalised patients[29,30]. These risk factors may be related to the ability of *A*. *baumannii* to colonise abiotic surfaces and medical devices, as well as the presence of comorbidities, the use of invasive procedures, and prior use of broad spectrum antibiotics[31–33]. Prior antibiotic exposures are frequently reported as a risk factor for MDR*A*. *baumannii* acquisition[3]. In our study, 82.9% of patients had previous exposure to carbapenems, and the use of cephalosporins resulted in a six-fold increased risk of acquiring OXA-23-producing *A*. *baumannii*.

This study provides important information on hospital epidemiology and infection control, specifically on the surveillance and monitoring of MDR pathogens. Factors such as age, comorbidities, invasive procedures, prolonged hospitalisation, and previous exposure to multiple antimicrobial classes may have influenced the high resistance rates and the spread of OXA-23-producing *A*. *baumannii* strains in the adult ICU [3,32,34]. The presence of associations between ISAba1/OXA-51 and ISAba1/OXA-23 was observed in all strains. Previous studies showed that coexistence of these resistance mechanisms can lead to high MDR phenotypes frequencies and high MICs for carbapenems[35–37].

MLST typing showed that ST1[9,11], ST15, ST25[9,29] and ST79[9,11], were the predominant genotypes. Clonal type ST79 appears to be the most important and disseminated clonal group, being detected throughout the study period, demonstrating the long-term persistence of this endemic clone responsible for nosocomial infections in ICUs. In Brazil, OXA-23-producing *A*. *baumannii* strains are mainly related to ST79, which is considered a high-risk clone in ICUs. Several outbreaks due to these strains have been reported in different Brazilian states, especially in South-eastern and Mid-western regions, which demonstrate a remarkable capacity for the dissemination and maintenance of this clone for many years in Brazilian hospitals[8,24,38–41]. ST1 corresponds to international clone I and, together with ST79 and ST15, these are the most frequently spread OXA-23-producing *A*. *baumannii* clones in Brazilian hospitals[8,24]. ST25 has been reported in Brazil more recently[24]. This finding is not surprising since Mato Grosso do Sul state shares a border with Bolivia, where *A*. *baumannii* strains belonging to this clonal group have previously been reported[25]. Interestingly, all patients in this study who had been infected or colonised with the ST25 OXA-23-producing *A*. *baumannii* strain died.

Through the implementation of infection control measures, a considerable reduction in the incidence of carbapenem-resistant *A*. *baumannii* was observed after November/2014. Transmission of closely related isolates between patients in the same ICU within a short period of time indicates the transmission of MDR strains from colonised or infected patients to healthcare staff, or vice versa. Contact isolation measures using single rooms with individual sanitary facilities have played a key role in controlling the circulation of carbapenem-resistant strains in the ICU and preventing future outbreaks[42].

Our findings show that OXA-23-producing *A*. *baumannii*, particularly ST79 (CC79), was associated with several risk factors and high mortality rates in ICU patients. With the emergence of OXA-23-producing *A*. *baumannii*, these results highlight the importance of reinforcing proper adherence to hospital infection control measures, particularly with patients transferred from other hospitals.
